# Cardiac tamponade complicating esophagectomy and retrosternal gastric tube reconstitution in a patient with an abnormal ascending aorta position: a case report

**DOI:** 10.1186/s40792-024-01850-9

**Published:** 2024-02-28

**Authors:** Shu Aoyama, Yasuhiro Miyazaki, Masaaki Motoori, Masashi Hirota, Takefumi Itami, Sayaka Matsumoto, Masataka Hirano, Michihiro Aomatsu, Takasumi Goto, Mutsunori Kitahara, Yuki Ozato, Yujiro Nishizawa, Hisateru Komatsu, Akira Inoue, Yoshinori Kagawa, Akira Tomokuni, Kazuhiro Iwase, Hiroyuki Nishi, Kazumasa Fujitani

**Affiliations:** 1 Department of Gastroenterological Surgery, Osaka General Medical Center, 3-1-56 Bandaihigashi, Sumiyoshi-ku, Osaka, Japan; 2 Department of Cardiovascular Surgery, Osaka General Medical Center, Osaka, Japan; 3 Department of Cardiovascular Surgery, Toyonaka Municipal Hospital, Toyonaka, Japan; 4 Department of Cardiovascular Surgery, Osaka National Hospital, National Hospital Organization, Osaka, Japan

**Keywords:** Esophageal cancer, Esophagectomy, Retrosternal gastric tube reconstitution, Cardiac tamponade

## Abstract

**Background:**

Cardiac tamponade is a rare postoperative complication of esophageal cancer surgery, which leads to rapid hemodynamic changes and can be fatal if not treated properly and promptly. Herein, we report a case of cardiac tamponade after thoracoscopic subtotal esophagectomy and retrosternal gastric tube reconstitution for esophageal cancer that was successfully treated with surgical drainage.

**Case presentation:**

An 86-year-old man with lower thoracic esophageal cancer underwent thoracoscopic subtotal esophagectomy and retrosternal gastric tube reconstitution. No intra-operative complications were observed. On the first postoperative day, tachycardia and hypotension were observed, and pericardial effusion was identified on computed tomography images. The patient was diagnosed with obstructive shock secondary to cardiac tamponade. As percutaneous puncture drainage was not possible due to the presence of a retrosternal gastric tube, pericardiotomy with a small left anterior thoracotomy was performed, and a large amount of hematogenous fluid was drained, which instantly improved circulation. On the second postoperative day, the patient showed decreased pulse pressure, and computed tomography revealed a residual and enlarged hematoma around the right ventricle. The patient underwent surgical drainage and another pericardiotomy with a small right anterior thoracotomy was performed to drain the hematoma. At this time, multiple injuries to the fatty tissue, epicardium, and myocardium with active bleeding were observed on the anterior surface of the right ventricle near the root of the pulmonary artery. In this patient, the ascending aorta ran further to the right and dorsal sides than usual, causing the anterior wall of the right ventricle near the root of the pulmonary artery to be closer to the back of the sternum. This abnormality may have contributed to injury during the creation of the retrosternal pathway, leading to cardiac tamponade.

**Conclusions:**

Cardiac tamponade after esophagectomy can occur because of manipulation during creation of the retrosternal route, with an anomaly in the aortic position being present in this case. Gentle manipulation and selection of the reconstruction route according to the patient’s condition are necessary in cases with such anatomical abnormalities.

## Background

Esophagectomy combined with preoperative chemotherapy or chemoradiotherapy is the standard treatment for locally advanced thoracic esophageal cancer [1]; however, esophagectomy is highly invasive and complications such as anastomotic leakage, pneumonia, recurrent nerve palsy, and arrhythmias, including atrial fibrillation, can occur [2]. Although cardiac tamponade is an extremely rare complication of esophagectomy [3], it can be promptly fatal and requires immediate treatment [4]. We herein report a case of cardiac tamponade caused by intrapericardial hemorrhage after thoracoscopic esophagectomy and retrosternal gastric tube reconstruction for thoracic esophageal cancer.

## Case presentation

An 87-year-old man was referred to our department for dysphagia that had started 2 months before. He presented hypertension, type 2 diabetes mellitus, and dyslipidemia as comorbidities, in addition to a history of smoking 20 cigarettes per day until the age of 45 years and drinking 1 unit of alcohol per day. The patient did not receive any antithrombotic therapy and was not taking any anticoagulant medication. He was 160 cm tall and weighed 70 kg. Blood tests revealed a mildly impaired renal function (creatinine, 1.01 mg/dL; creatinine clearance, 51.02 mL/min), impaired glucose tolerance (hemoglobin A_1c_ level, 6.6%), and mild anemia (hemoglobin, 12.0 g/dL).

Upper gastrointestinal endoscopy revealed an elevated lesion 31–36 cm from the incisors, and advanced esophageal cancer was suspected, with a biopsy revealing a squamous cell carcinoma. Contrast-enhanced computed tomography (CT) showed wall thickening of the lower thoracic esophagus. Involvement of the surrounding organs was not evident, and the tumor invasion depth was determined to extend until the esophageal adventitia. One enlarged lymph node was found near the left main bronchus; however, no distant metastases or other non-resectable factors were identified, and the lesion was deemed resectable. Cardiac and respiratory functions were within the normal range, and the patient had an Eastern Cooperative Oncology Group performance status of 0; therefore, we opted for esophagectomy as the treatment modality. Owing to the patient’s age and renal impairment, neoadjuvant therapy was not administered.

The patient underwent McKeown operation with subtotal thoracic esophagectomy (with a thoracoscopic right thoracic approach), two regional lymph node dissections, hand-assisted laparoscopic narrow gastric tube creation, reconstruction with a retrosternal narrow gastric tube, and jejunostomy (for nutrition). The operation time was 12 h 20 min, with a blood loss of 100 mL. The retrosternal pathway was sharply dissected from the abdominal wound with an electrocautery scalpel using an endoscope, followed by blunt dissection cephalad along the back of the sternum with a spatula and continued to the cervical wound, creating enough retrosternal space for the narrow gastric tube with a width of 3.5 cm. The retrosternal pathway was created without hemorrhage or other complications, and no intraoperative changes in circulatory dynamics were observed. No intraoperative complications were observed. Postoperatively, the patient was admitted to the intensive care unit unextubated and unawakened.

**Fig. 1:**
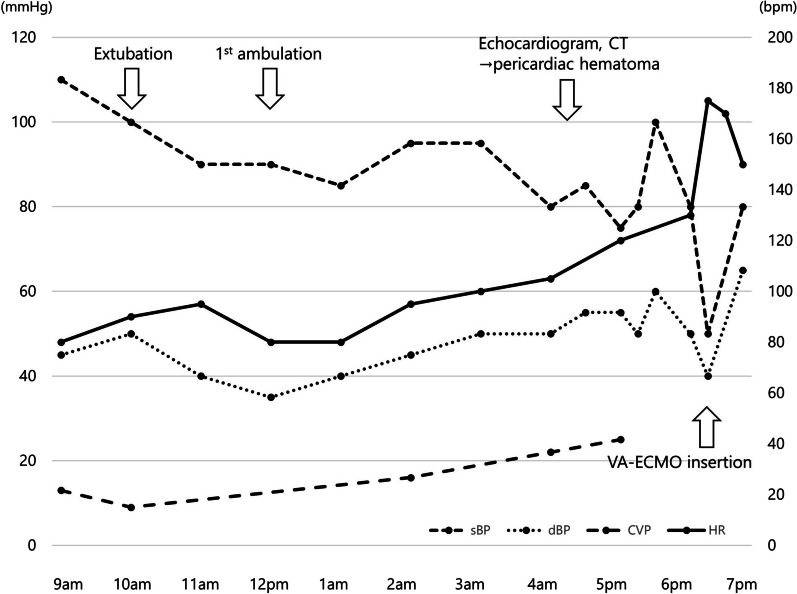
The time course and trends of vital signs on the first postoperative day. *CT* computed tomography, *VA-ECMO* venoarterial extracorporeal membrane oxygenation, *sBP *systolic blood pressure, *dBP *diastolic blood pressure, *CVP *central venous pressure, *HR* heart rate

**Fig. 2:**
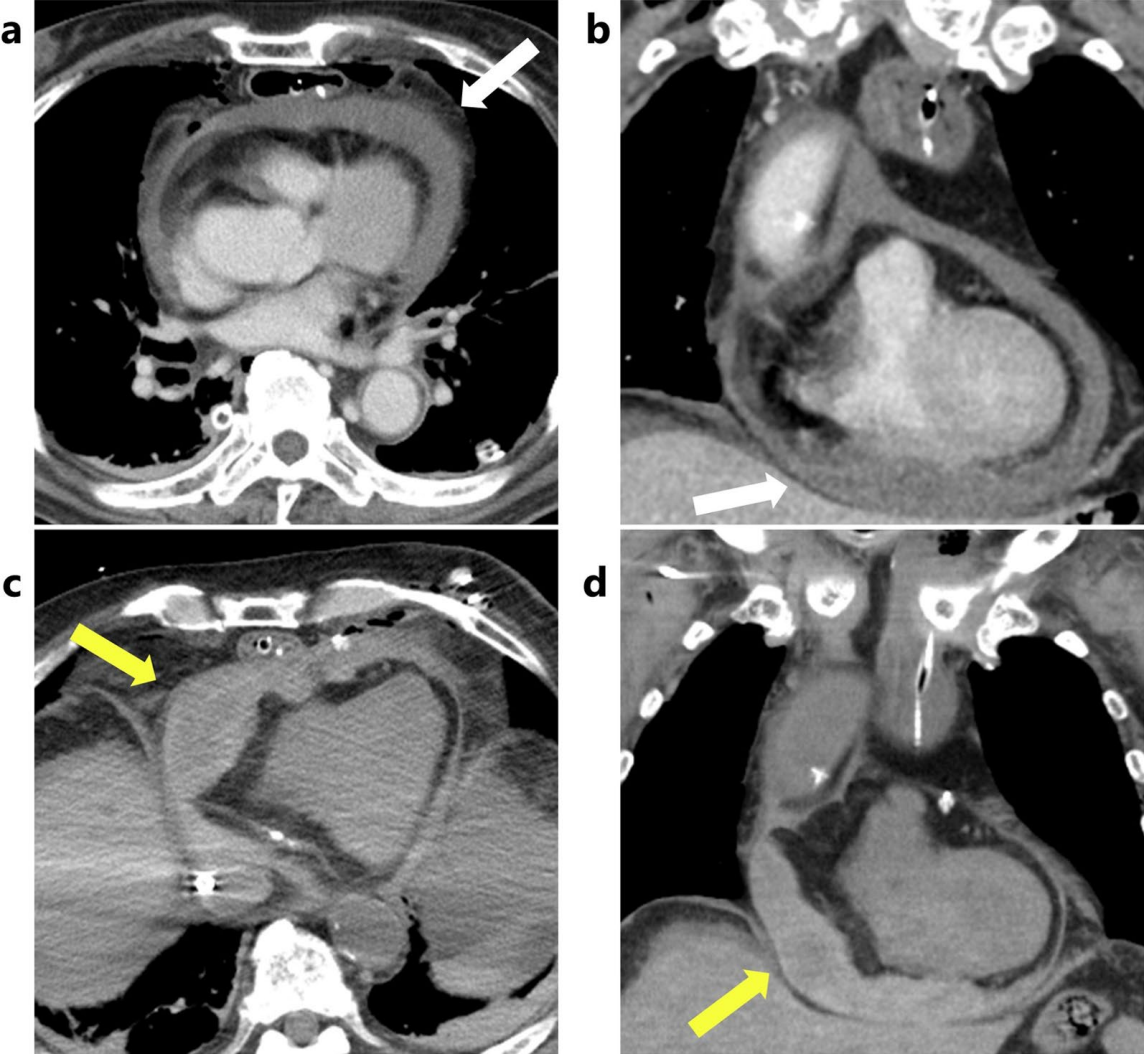
Computed tomography images. **a**, **b** Images before the first drainage showed a high-density fluid accumulation in the pericardium (white arrows). **c**, **d** Images before the second drainage showed an enlarged residual hematoma around the right ventricle (yellow arrows)

The time course on the first postoperative day is shown in Fig. 1. The patient's initial postoperative course was stable and he was extubated on the morning of the first postoperative day. His first postoperative ambulation occurred 2 h after extubation. However, approximately 5 h after extubation, a gradual increase in heart rate, decrease in blood pressure, and increase in central venous pressure were observed. After 1 h, his blood pressure dropped significantly, requiring catecholamine administration. Imaging studies were then performed approximately 7 h after extubation, and echocardiography revealed a pericardial effusion—due to the presence of the gastric tube, only the apex of the heart was observable. Contrast-enhanced CT showed a collection of high-concentrate pericardial fluid, suggesting a cardiac tamponade caused by the intrapericardial hematoma leading to obstructive shock (Figs. 2a, b). Echo-guided pericardiocentesis via the subxiphoid approach was not possible because of the presence of the retrosternal reconstructed gastric tube, which prevented the access route. Approximately 8 h after extubation, while the treatment modalities were being debated by the medical staff, circulatory disturbance due to atrial fibrillation occurred, and cardiac extracorporeal membrane oxygenation was initiated. Heparinization was not performed because of the possibility of exacerbating intrapericardial hemorrhage. Because puncture drainage was not possible, surgical drainage was performed.

**Fig. 3:**
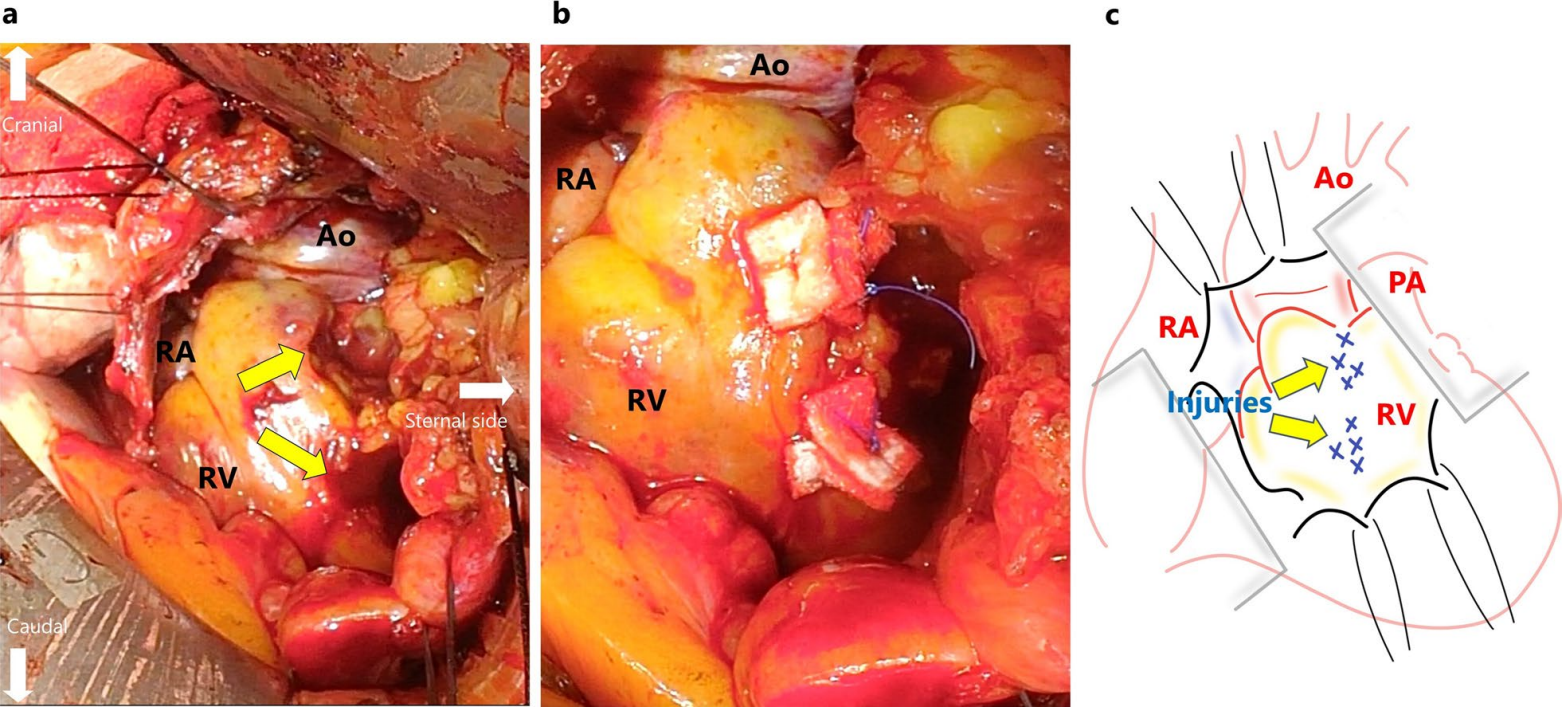
Pictures from the second surgical drainage. **a** The fatty tissue, endocardium, and myocardium were injured on the anterior surface of the right ventricle (yellow arrows), and oozing from such was observed. **b** The damaged sites were suture-repaired. **c** Overall schema. *Ao* aorta, *RA* right atrium, *RV* right ventricle, *PA* pulmonary artery

Because the patient presented a retrosternal gastric tube between the sternum and heart, a median sternotomy was avoided to preserve it. A small left anterior thoracotomy in the fourth intercostal space and subsequent pericardiotomy were performed. Upon opening of the pericardium, approximately 600 mL of bloody effusion was drained, resulting in instant hemodynamic improvement. No bleeding points were detected inside the pericardium on endoscopic observation. No persistent bleeding from the pericardium remained, and the surgery ended with the placement of a pericardial drainage tube in the pericardium. However, his blood pressure dropped again the following day, and CT revealed an enlarged residual hematoma in the pericardium around the right ventricle (Fig. 2c, d). We decided to perform another surgical drainage to improve the patient's hemodynamics and wean him off cardiac extracorporeal membrane oxygenation. In this second procedure, we approached the pericardium through a small right anterior thoracotomy in the fourth intercostal space to remove the hematoma around the right ventricle. After pericardiotomy, approximately 100 mL of the hematoma was removed from the right ventricular perimeter. Endoscopic observation was performed again and observation of the anterior aspect of the right ventricle, which could not be evaluated during the first surgical drainage, revealed injuries to the fatty tissue, epicardium, and myocardium of the right ventricular wall near the root of the pulmonary artery, with persistent oozing being detected (Fig. 3a). Such sites were suture-repaired and hemostasis was confirmed (Fig. 3b). The injury sites were located intermittently along the posterior side of the sternum, in the craniocaudal direction. As no intraoperative procedure other than the creation of the retrosternal pathway could have caused such injury, we considered that it occurred bluntly during that procedure. After the second surgical drainage, the patient’s circulation improved and he was weaned off cardiac extracorporeal membrane oxygenation on the fourth postoperative day. Thereafter, due to the presentation of right heart failure, surgical drainage was performed again, but there were no bleeding points, and the remaining hematoma was removed as much as possible. Hemostasis was considered achieved during the second surgical drainage procedure. Then, the patient did not present with any symptoms of cardiac tamponade.

Acute renal failure, pleural effusion associated with circulatory changes, and postoperative atrial flutter requiring ablation therapy occurred, and improvement in the respiratory status was slow. Although tracheotomy was performed on the 10th day after esophageal cancer surgery, the patient was weaned from the respirator nearly a month after the operation. No anastomotic leakage was observed. After swallowing training, the patient started oral intake on the 31st postoperative day. Although he was hospitalized for a long time because of a pyothorax, his general condition gradually improved, and he was transferred to another hospital for rehabilitation on the 120th postoperative day.

The histopathological diagnosis of the esophageal tumor was a spindle cell squamous cell carcinoma with invasion of the lamina propria mucosa—most of the lesion was thought to have detached at the time of the operation because of necrosis—and no lymph node metastasis was detected. Resection margins were negative. Adjuvant therapy was not administered. The patient had a recurrence-free course, but died 18 months after surgery due to pneumonia.

## Discussion

In this report, we have described a case of cardiac tamponade caused by an intrapericardial hemorrhage in the early postoperative period after esophageal cancer surgery, which was successfully treated with surgical drainage. Cardiac tamponade is an especially rare complication after esophageal cancer surgery [3]; however, in this case, the patient's anatomical anomaly of the aortic position was considered to have contributed to the right ventricular injury, resulting in cardiac tamponade.

The incidence of cardiac tamponade after esophagectomy has been reported to be 0.74% [3]. In PubMed, only 8 cases of cardiac tamponade at the early period after esophagectomy and reconstruction have been yet reported [as searched with the keywords ("oesophagectomy" OR "esophagectomy") AND "tamponade"; and excluding cases of transhiatal esophagectomy; Table 1]. Two types of cardiac tamponade after esophagectomy can occur. The first is due to extra-pericardial compression, which, strictly speaking, is not a cardiac tamponade and has been reported to be caused by dilatation of the reconstructed gastric tube [5, 6] and chylothorax [7]. The second is caused by fluid collection in the pericardium, and has been reported to be caused by acute endocarditis [8], chylous fistula of the pericardium [9, 10], and intrapericardial hemorrhage, as in the present case [4, 11]. All cited cases were managed with percutaneous or surgical drainage, suggesting that, although cardiac tamponade is potentially fatal, it can be successfully managed with prompt treatment.

**Table 1:** Reported cases of early cardiac tamponade after esophagectomy

No.	Year	First Author	Location of tumor	Operative procedure for esophagectomy	Reconstruction organ	Reconstruction route	Date of cardiac tamponade	Cause of tamponade	Treatment	Outcome	Reference
1	2001	Cherian	NA	NA	Gastric tube	NA	POD 1	Dilation of gastric tube	Suction of gastric tube	Alive	[5]
2	2001	Kariya	Lt	Right thoracotomy	Gastric tube	Retrosternal	POD 1	Dilation of gastric tube	Suction of gastric tube	Alive	[6]
3	2012	Rottoli	Lt	Right thoracotomy	Gastric tube	Posterior mediastinal	POD22	Chylothorax	Transabdominal drainage	Alive	[7]
4	1998	Levitt	EGJ	Left thoracoabdominal	NA	NA	POD 0	Intrapericardial hemorrhage	Open drainage from the scar of esophagectomy	Alive	[11]
5	2005	Mizuguchi	Ut	Right thoracotomy	Gastric tube	Retrosternal	POD 4	Acute pericarditis	Percutaneous drainage via the subxiphoid	Alive	[8]
6	2015	Ito	Lt	Right thoracotomy	Gastric tube	Retrosternal	POD4	Intrapericardial hemorrhage	Open drainage with left anterior thoracotomy	Alive	[4]
7	2017	Kosugi	Mt	Right thoracotomy	Gastric tube	Retrosternal	POD1,13	Chylopericardium	Percutaneous drainage via the subxiphoid	Alive	[9]
8	2020	Li	Mt	Minimal right thoracotomy	Gastric tube	NA	POD1	Chylopericardium	Percutaneous drainage via the subxiphoid	Alive	[10]
9	2023	Aoyama	Lt	Right thoracoscopy	Gastric tube	Retrosternal	POD1	Intrapericardial hemorrhage	Open drainage with left and right anterior thoracotomy	Alive	–

Cases 1–3 occurred due to pressure from outside the pericardium, whereas cases 4–9 occurred due to fluid collection in the pericardium

**Fig. 4:**
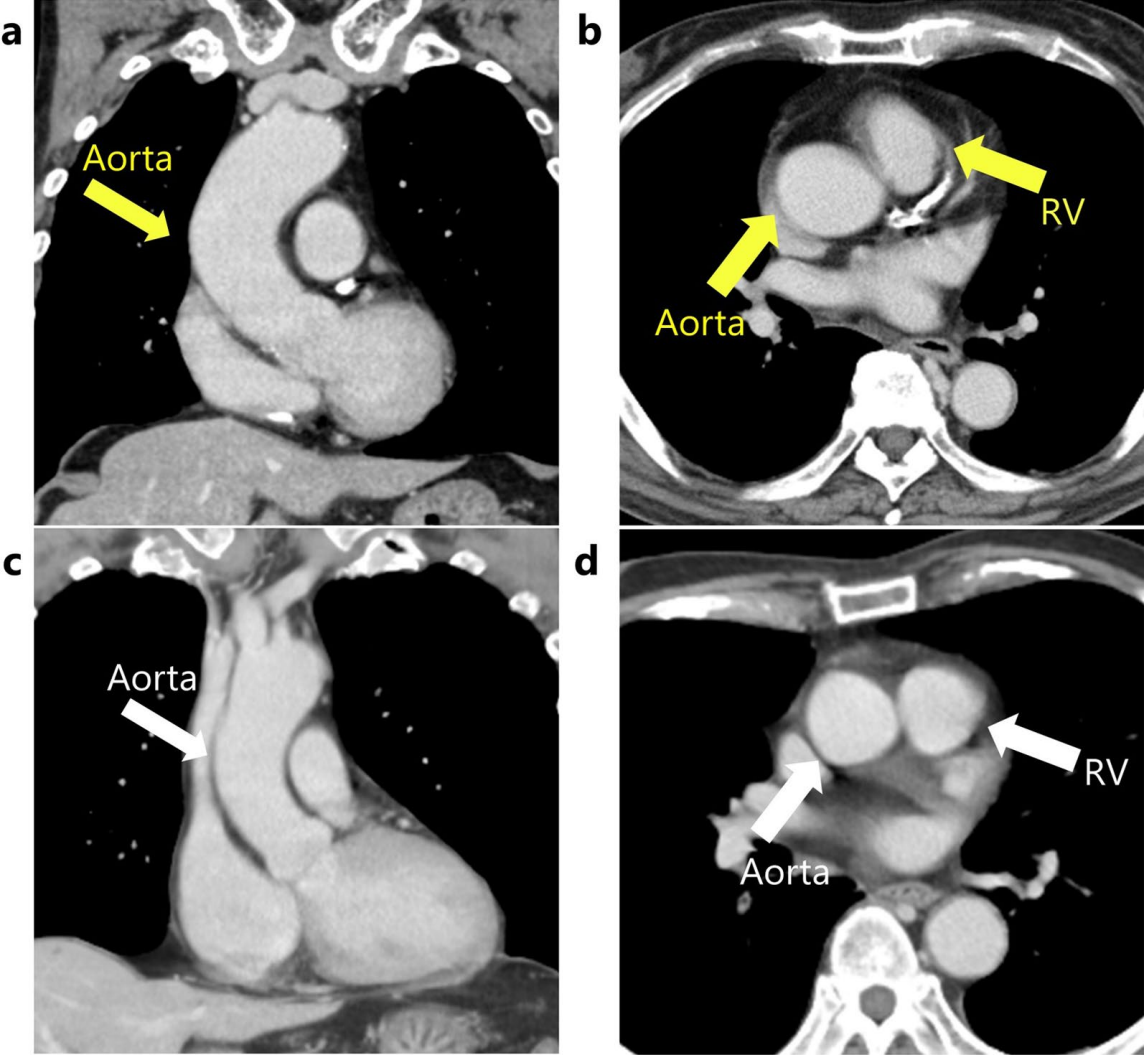
Preoperative computed tomography images. **a**, **b** The ascending aorta ran dorsally and more curved to the right than usual, and the right ventricle near the root of the pulmonary artery rose toward the sternum. **c**, **d** Images of a normal anatomy. *RV* right ventricle

In the present case, lesions to the fatty tissue, epicardium, and myocardium of the right ventricle caused by blunt injury during the creation of the retrosternal pathway were thought to have resulted in cardiac tamponade due to intrapericardial hemorrhage. We suspect that the reason for the occurrence of these injuries in this case was an anatomical anomaly. Preoperative CT showed that the ascending aorta was markedly more curved to the right dorsal side than usual, which caused the anterior wall of the right ventricle near the pulmonary artery root to be located just below the sternum (Fig. 4). In general, the left ventricular wall has a thicker and stronger muscular layer than the right ventricular wall [12], and the ascending aorta is thicker and more robust than the pulmonary artery [13]. Although the anterior aspect of the ascending aorta and left ventricle are usually located behind the sternum, in the present case, the anterior wall of the right ventricle near the root of the pulmonary artery, which is more vulnerable, was located behind the sternum. This anatomical abnormality may have contributed to the injuries that occurred during the creation of the retrosternal pathway. Although previous cases of cardiac tamponade due to intrapericardial hemorrhage after esophagectomy and reconstruction with a retrosternal gastric tube have been reported [4], the etiology of the hemorrhage was unknown and solely suggested to have occurred during thoracic esophagectomy. This is the first reported case of intrapericardial hemorrhage leading to cardiac tamponade caused by manipulation during gastric tube reconstruction. In the present case, a narrow gastric tube was used and the retrosternal dissection was performed gently, avoiding excessive dissection. However, intrapericardial hemorrhage still occurred. In addition to the anatomical anomaly, arteriosclerosis caused by hypertension, dyslipidemia, and aging, as well as the abundance of intrapericardial fat, which was one of the sources of hemorrhage, may have contributed to the intrapericardial hemorrhage. Patients with anatomical abnormalities, such as in this case, those who are undergoing antithrombotic therapy, have fragile vessels due to atherosclerosis, have a small thoracic cage and a short distance between the sternum and pericardium, or are obese with abundant intrapericardial fat may be at risk of intrapericardial hemorrhage during the retrosternal route creation. Therefore, careful and gentle manipulation is required in such cases, and selecting a reconstruction route other than the retrosternal one may be desirable depending on the patient’s condition.

Percutaneous puncture drainage, which is less invasive than surgical drainage, is usually performed to treat cardiac tamponade [14]; however, obtaining a puncture route may be difficult in cases in which a gastric tube is present in the retrosternal pathway. In previous reports of cardiac tamponade after reconstruction with a retrosternal gastric tube, puncture drainage via the subxiphoid approach was possible in some cases [8, 9], whereas in others, as in our case, puncture drainage was not feasible, and surgical drainage with a small thoracotomy was performed [4]. As an approach for surgical drainage of cardiac tamponades, especially in emergency settings, left anterior thoracotomy is reportedly easy and safe because of its proximity to the pericardium [15, 16]; therefore, a left anterior thoracotomy was chosen for the initial surgical drainage in the present case. However, because the bleeding points were located at the right ventricular wall, such could not be detected during the first surgical drainage, and the hematoma remained and expanded, resulting in recurrent cardiac tamponade, which required another surgical drainage with a right anterior thoracotomy. During the first surgical drainage, additional observation through the right anterior thoracotomy may have prevented another surgical drainage. In addition, some cases of open-heart surgery with median sternotomy being performed in cases with a retrosternal gastric tube have been reported [17]. Although more invasive and potentially damaging to the gastric tube, a median sternotomy approach might have been an optional technique for draining the pericardium entirely.

In this report, we described a case of early postoperative cardiac tamponade after esophageal cancer surgery due to intrapericardial hemorrhage from an injury to the right ventricular wall caused during retrosternal pathway creation in a patient with an abnormal aortic anatomy, which was successfully treated by surgical drainage. Cardiac tamponade may have been prevented by preoperatively investigating for anatomical abnormalities. Additionally, in cases in which cardiac tamponade occurs after reconstruction with a posterior sternal gastric tube, puncture drainage should be attempted, and surgical drainage should be prepared at the same time. The primary approach for surgical drainage is a left anterior thoracotomy; however, if the tamponade is caused by intrapericardial hemorrhage and the bleeding point cannot be identified, performing a right anterior thoracotomy simultaneously might be necessary to identify the bleeding point and achieve hemostasis.

## Conclusions

A case of cardiac tamponade after esophagectomy and retrosternal gastric tube reconstruction was successfully treated with surgical drainage. Cardiac tamponade can occur because of ventricular injury caused by manipulation during retrosternal route creation, and an anomaly in the aortic position was present in this case. In such cases, careful and gentle manipulation is required, and the selection of a reconstruction route other than the retrosternal one might be desirable.

## Data Availability

The authors declare that the data used in this study can be made available upon any reasonable request.

## Abbreviation

CT: Computed tomography
